# Natural amines inhibit activation of human plasmacytoid dendritic cells through CXCR4 engagement

**DOI:** 10.1038/ncomms14253

**Published:** 2017-02-09

**Authors:** Nikaïa Smith, Nicolas Pietrancosta, Sophia Davidson, Jacques Dutrieux, Lise Chauveau, Pasquale Cutolo, Michel Dy, Daniel Scott-Algara, Bénédicte Manoury, Onofrio Zirafi, Isabelle McCort-Tranchepain, Thierry Durroux, Françoise Bachelerie, Olivier Schwartz, Jan Münch, Andreas Wack, Sébastien Nisole, Jean-Philippe Herbeuval

**Affiliations:** 1 CNRS UMR-8601, Université Paris Descartes, CICB, 45 rue des Saints-Pères, 75006 Paris, France; 2 Team Chemistry & Biology, Modeling & Immunology for Therapy, CBMIT; 3 Immunoregulation Laboratory, Francis Crick Institute, London NW1 1AT, UK; 4 INSERM UMR-S 1124, Université Paris Descartes, 45 rue des Saints-Pères, 75006 Paris, France; 5 Institut Pasteur, Virus & Immunity Unit, 25-28 Rue du Dr Roux, Paris 75015, France; 6 Inflammation Chimiokines et Immunopathologie, INSERM, Faculté de médecine—Université Paris-Sud, Université Paris-Saclay, 92140 Clamart, France; 7 CNRS UMR-8147, Hôpital Necker, 156 Rue de Vaugirard, 75015 Paris, France; 8 Institut Pasteur, Unité de Régulation des Infections Rétrovirales, 25-28 Rue du Dr Roux, Paris 75015, France; 9 INSERM U1151, CNRS8253, Université Paris Descartes, Hôpital Necker, 156 Rue de Vaugirard, 75015 Paris, France; 10 Institute of Molecular Virology, Ulm University Medical Center, Ulm 89081, Germany; 11 Institut de Génomique Fonctionnelle, CNRS UMR5203, INSERM U661, Université de Montpellier (IFR3), 141 rue de la Cardonille, F-34094 Montpellier Cedex 5, France

## Abstract

Plasmacytoid dendritic cells (pDC) are specialized in secretion of type I interferon in response to pathogens. Here we show that natural monoamines and synthetic amines inhibit pDC activation by RNA viruses. Furthermore, a synthetic analogue of histamine reduces type I interferon production in a mouse model of influenza infection. We identify CXC chemokine receptor 4 (CXCR4) as a receptor used by amines to inhibit pDC. Our study establishes a functional link between natural amines and the innate immune system and identifies CXCR4 as a potential ‘on-off' switch of pDC activity with therapeutic potential.

## Abstract (web summary)

Plasmacytoid dendritic cells produce type I interferons in response to viral sensing. Here the authors show that amines inhibit these plasmacytoid dendritic cell responses through CXCR4 engagement.

Plasmacytoid dendritic cells (pDC) are the first line of host defence against viruses and bacteria^1^ and link innate to adaptive immunity^2^. These immune cells are activated after recognition of pathogen nucleic acids by sensors such as Toll-like receptors (TLR). pDC express TLR7 and TLR9 (refs 3, 4), which respond to single stranded RNA (ssRNA) and imidazoquinolines^5^,^6^ or DNA and CpG-containing oligonucleotides^7^, respectively. TLR activation triggers production of type I interferon and proinflammatory cytokines^8^ through MyD88-mediated IRF7 signalling. RNA viruses, such as human immunodeficiency virus type 1 (HIV-1) and dengue virus, induce secretion of type I interferon and membrane expression of the proapototic ligand TNF-related apoptosis inducing ligand (TRAIL), a TNF superfamily member, via the TLR7 pathway, transforming pDC into interferon-producing killer pDC (IKpDC)^9^,^10^,^11^. In mice, IKpDC induce tumour cell apoptosis^12^. In addition, prolonged pDC activation and consequently massive type I interferon production may have adverse effects in autoimmune diseases and the chronic phase of AIDS^13^,^14^,^15^. Furthermore, type I interferon overproduction is also linked to immunopathology in acute viral infections such as influenza^16^. Therefore, modulating pDC function and understanding the mechanisms underlying this pDC regulation is of great clinical interest.

The modulation of pDC activation is only partly documented. Synthetic molecules such as chloroquine^10^, rapamycin^17^ or anti-BDCA-2 antibodies^18^ decrease type I interferon production through various mechanism. The effect of natural molecules, such as amines, on pDC regulation is not yet characterized. Natural amines are small positively charged molecules that have an essential function in various cellular functions. For example, dopamine and serotonin are key neurotransmitters in the central nervous system, and histamine is involved in allergy response. Natural amines may directly interact with immune cells by modulating their activation. Histamine strongly inhibits cytokine production by Influenza A-activated pDC (ref. 19) and inhibits type I interferon production by activated pDC from psoriasis patients, probably through the histamine receptor 4 (H4R)^20^. The atopic phenotype in children, characterized by hyper-histamine secretion, is associated with a reduction in virus-induced interferon-α (IFN-α) release^21^.

Here we show that natural and synthetic monoamines and polyamines inhibit type I interferon production, membrane TRAIL expression and interferon-stimulating genes (ISG) by virus-stimulated pDC and PBMCs *in vitro* and with a mouse model of influenza infection. Surprisingly, histamine receptors are not required for pDC inhibition. We show that the positive ammonium moiety is essential for the inhibitory activity and we identify CXCR4 as the unexpected common link between amine effect and pDC inhibition. Our study identifies CXCR4 as a potential modulator of pDC activity and therefore as a promising therapeutic target in autoimmune diseases and chronic infections.

## Results

### Histamine and its analogue inhibit pDC activation

**Figure 1:**
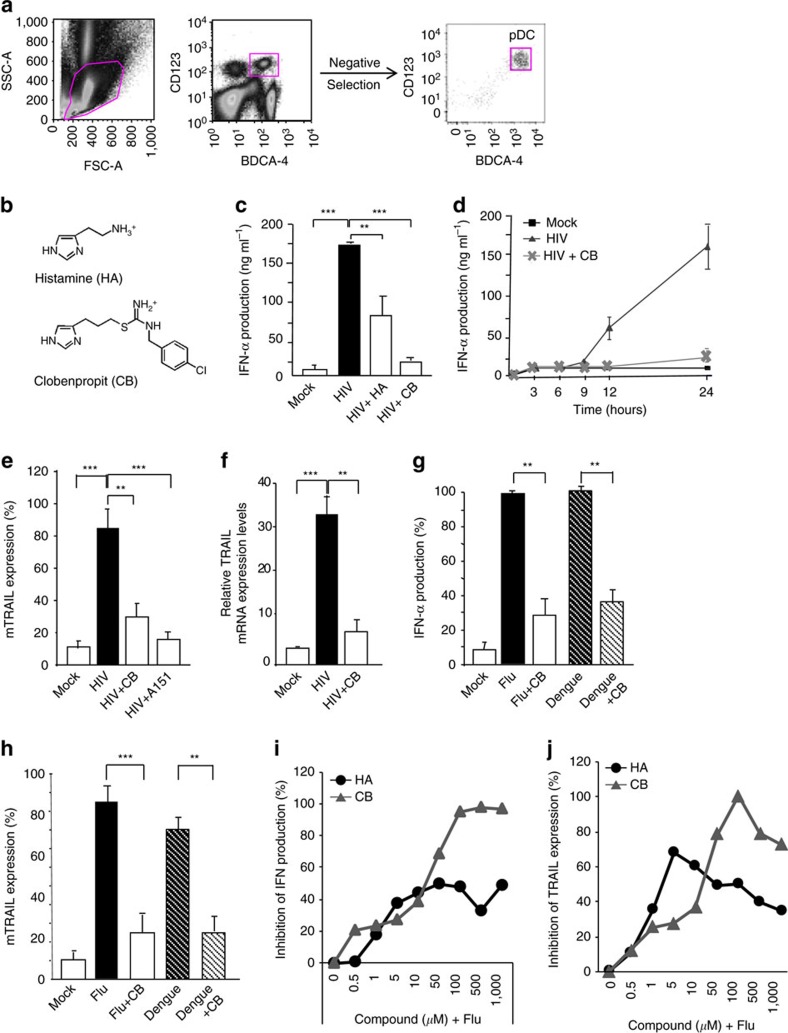
Histamine and Clobenpropit inhibit purified HIV-stimulated pDC activation. (**a**) pDC were acquired by flow cytometry before and after purification from PBMC of healthy donors. Negative selection was used to obtain purity higher than 90% in all experiments. (**b**) Chemical structures of histamine (HA) and clobenpropit (CB). (**c**) Isolated pDC were pretreated with histamine or with CB at the concentration of 10 μM and then stimulated with microvesicles (mock) alone or with HIV overnight. IFN-α was measured in the supernatants by Elisa. (**d**) IFN-α production overtime was measured in supernatants of purified pDC cultured overnight with microvesicles (mock) alone or with HIV alone or together with CB at the concentration of 10 μM. (**e**) Levels of membrane TRAIL were assessed by flow cytometry on purified pDC after pre-incubation with CB or the specific TLR7 antagonist, A151, and then overnight HIV stimulation. (**f**) mRNA levels of TRAIL from purified pDC pre-incubated with CB (10 μM) and stimulated overnight with HIV, were measured by RT-qPCR and normalized to RPL13A. Production of IFN-α by Elisa in supernatants (**g**) or expression of membrane TRAIL at the surface of the cells by flow cytometry (**h**) from purified pDC pre-incubated with CB and then cultured overnight with Influenza A or Dengue viruses. Inhibition of IFN-α production (**i**) and inhibition of TRAIL expression (**j**) were assessed in supernatants of PBMC and by flow cytometry on PBMC cultured overnight with Influenza A virus alone or together with histamine (HA) or CB at a concentration ranging from 0.5 μM to 1 mM. Data shown as the mean of three independent experiments ±s.e.m. *P* values (*p*) were determined using a two-tailed Student's *t* test. ***P<0.001, **P<0.01, *P<0.05.

As histamine has been shown to inhibit Influenza A virus (Flu)-induced pDC activation^19^, we examined its effect on pDC activation by HIV-1. A dose range analysis indicated that histamine was active at 10 μM on purified pDC (Fig. 1a; Supplementary Fig. 1a) without obvious toxicity (Supplementary Fig. 1b). A study showed that the histamine receptor 4 (H4R) was responsible for the inhibitory effect of histamine on human pDC (ref. 20). Thus, we tested the effect of the H4R agonist clobenpropit (CB) (Fig. 1b). CB showed a stronger inhibitory effect than histamine, and reduced levels of IFN-α secreted following HIV-1 stimulation by ∼90% (Fig. 1c). Furthermore, CB had no cytotoxic effect at the concentration of 10 μM (Supplementary Fig. 1b). We next assessed IFN-α production kinetics. CB inhibited IFN-α production by HIV-stimulated pDC right when IFN-α was secreted (Fig. 1d). CB showed similar inhibitory effect to a TLR-7 antagonist, A151 (Fig. 1e). Relative TRAIL messenger RNA(mRNA) expression levels were assessed by RT-qPCR and confirmed these results (Fig. 1f). CB also strongly inhibited IFN-α production (Fig. 1g) and membrane TRAIL expression (Fig. 1h) by pDC cultured with Influenza and Dengue viruses, demonstrating that CB effect was not restricted to HIV-1. We then tested endogenous and synthetic amines in a range from 5.10^−7^ to 10^−3^ M on type I interferon production (Fig. 1i) and membrane TRAIL expression (Fig. 1j) on human Influenza-activated primary PBMC. Furthermore, we simultaneously studied cell viability under the several concentrations of amines (Supplementary Fig. 1b). Amines optimal effects were observed between 10^−5^ and 5.10^−5^ M for the clear majority of the molecules. Higher concentrations induced high levels of cell death (Table 1).

### Histamine receptors are not involved in inhibition of pDC

**Figure 2:**
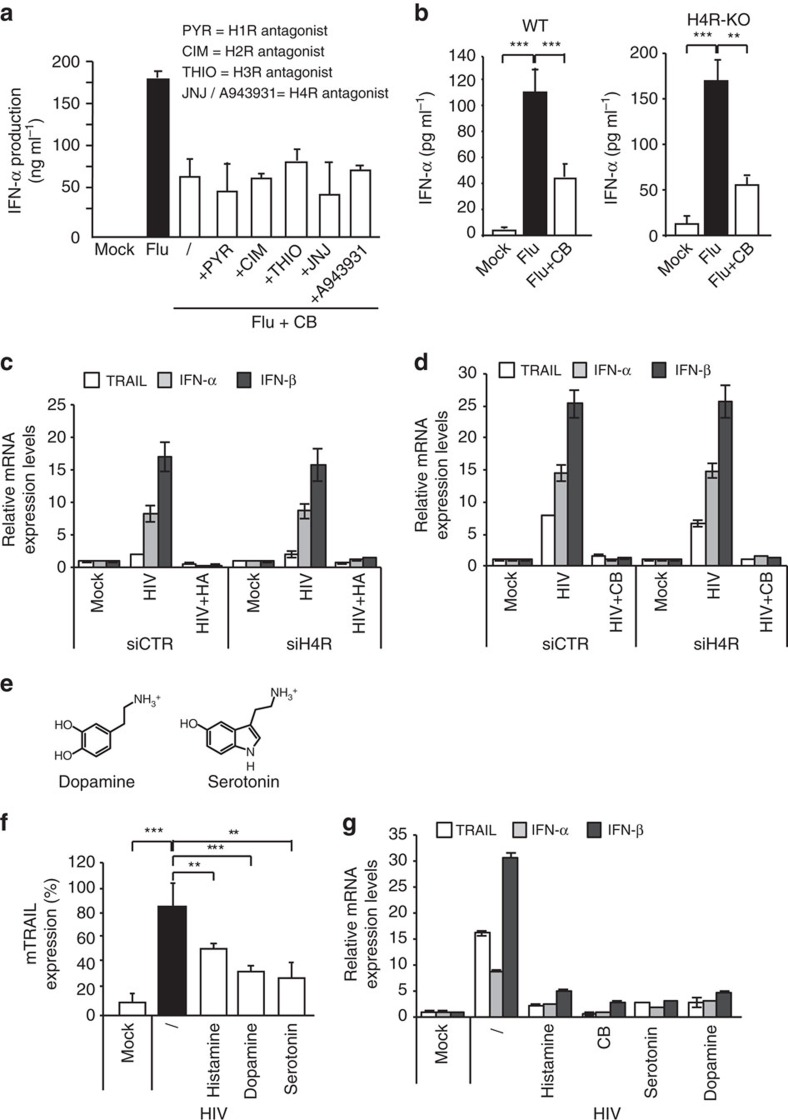
Histamine receptors are not involved in the inhibition of stimulated pDC. (**a**) Isolated pDC were pretreated for 1 h with various histamine receptors inhibitors, pyridylethylamine (H1R), cimedine (H2R), thioperamide (H3R) and JNJ7777120 and A943931 (H4R) at 50 μM, and then treated with CB before overnight stimulation with Influenza A virus. IFN-α was quantified in the supernatants by Elisa. (**b**) Mouse MNC (multinucleated cells) were obtained from the spleen using a homogenizer and purified using a 35% isotonic Percoll density gradient (Amersham Biosciences). Spleen MNC were depleted of RBC using red cell lysis buffer (8.3 mg ml^−1^ NH_4_Cl, 1 mg ml^−1^ KHCO_3_, and 3.72 μg ml^−1^ EDTA put in Mat and Med). Wild type (WT) (*n*=14) or H4RKO mice (*n*=10) spleen MNC were pre-incubated with CB (10 μM) and then cultured overnight with Influenza A virus. IFN-α was quantified in the supernatants by ELISA. *N*=5. (**c**) and (**d**) pDC were treated for 24 h with a siRNA H4R (siH4R) or a siRNA Control (siCTR) at 160 nM. Cells were pre-incubated with histamine (**c**) or CB (**d**) (10 μM) and then stimulated with HIV. mRNA levels of TRAIL, IFN-α and IFN-β from those cells were then quantified by RT-qPCR. (**e**) Chemical structures of histamine, dopamine and serotonin. (**f**) Levels of membrane TRAIL were assessed by flow cytometry on purified pDC after pre-incubation with histamine, dopamine and serotonin then overnight HIV stimulation. (**g**) mRNA levels of TRAIL and IFN-(α, β) from purified pDC pre-incubated with histamine, CB, dopamine and serotonin and stimulated overnight with HIV, were measured by RT-qPCR and normalized to RPL13A. When not specify, data shown as the means of three independent experiments ±s.e.m. *P* values (*p*) were determined using a two-tailed Student's *t* test. ***P<0.001, **P<0.01, *P<0.05.

Activation of histamine receptors has been described to block interferon production by virus-activated pDC (ref. 19). More recently, it has been shown that in psoriasis patients histamine inhibits type I interferon and cytokine production by pDC through histamine receptor 4 (H4R)^20^. We thus tested whether the activity of CB was dependent on histamine receptors. We evaluated CB in the presence of different histamine receptor antagonists (pyrilamine/PYR for H1R, cimetidine/CIM for H2R, thioperamide/THIO for H3R and JNJ7777120/JNJ or A943931 compounds for H4R at 10 μM on Influenza-stimulated pDC (Fig. 2a). Even in the presence of these antagonists, CB inhibited the IFN-α production triggered by influenza. To confirm these results, we stimulated pDC isolated from wild type (WT) or H4R knock-out (KO) mice with Flu after pre-incubation with CB and histamine. As HIV is unable to induce type I interferon or TRAIL expressions in mouse pDC because mouse pDC do not express the HIV coreceptor CD4, which is essential for pDC recognition and activation^22^, we used Flu to stimulate mice cells. We found that CB inhibited IFN-α production by Influenza-stimulated pDC from both wild type and H4R KO mice (Fig. 2b). Next, we silenced H4R in human primary pDC by siRNA, (Supplementary Fig. 1c) and then determined the effect of histamine and CB. We found that H4R knock down neither block histamine nor CB inhibitory activity on IFN-α, IFN-β and TRAIL productions by HIV-stimulated pDC. Thus, H4R is not implicated in our model of pDC modulation by histamine or CB, suggesting an alternative mechanism (Fig. 2c,d).

As histamine and CB are both amines, we thus examined whether amines in general display an inhibitory effect on pDC activation and analysed natural amines dopamine and serotonin (Fig. 2e). All amines inhibited HIV-mediated upregulation of membrane TRAIL and HLADR, and also migration and maturation markers such as CCR7, CD40, CD86 and CD80 expression (Fig. 2f; Supplementary Fig. 1d), as well as TRAIL, IFN-α/β mRNA by HIV-stimulated pDC (Fig. 2g). Notably, none of these molecules were cytotoxic at concentration used (Supplementary Fig. 1e). We also tested these amines alone on human primary pDC culture. As positive control we used HIV-1 to stimulate cytokine production by pDC. Membrane TRAIL and HLADR, as well as migration and maturation markers such as CCR7, CD40, CD86 and CD80 expression were not affected by the amines in the absence of HIV (Supplementary Fig. 1f). IFN-α, IFN-β and TRAIL mRNA expressions were quantified by RT-PCR and showed that none of the amines tested had an effect on type I interferon production in the absence of stimulation (Supplementary Fig. 1g).

### HA and CB inhibit virus-induced IFN in PBMC and mice

**Figure 3:**
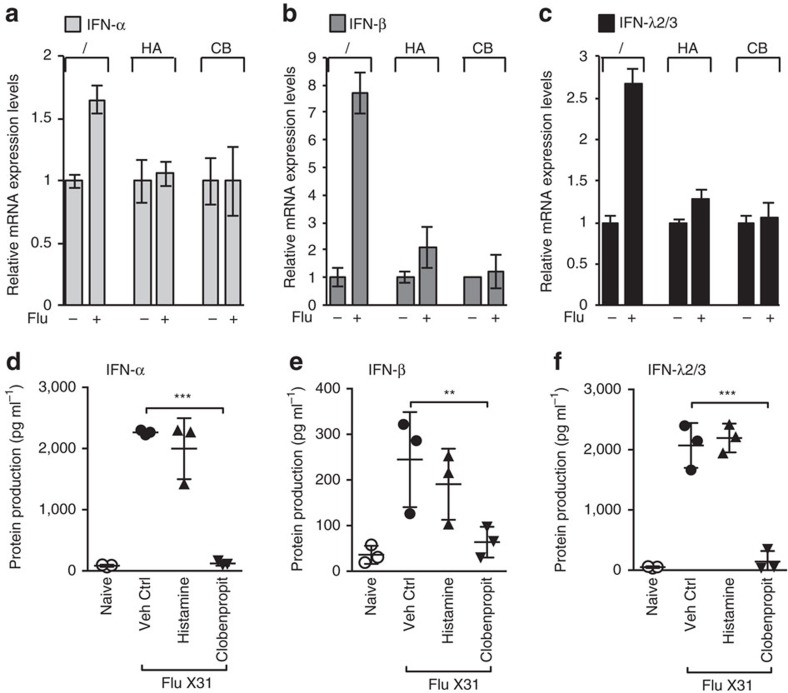
HA and CB inhibit Influenza-induced production of type I interferons in PBMC and mice. (**a**–**c**) mRNA levels of IFN-α (**a**), IFN-β (**b**) and IFN-λ2/3 (**c**) from PBMC pre-incubated with histamine, and CB and stimulated overnight with Influenza, were measured by RT-qPCR and normalized to RPL13A. Data shown as the means of three independent experiments ±s.e.m. (**d**–**f**) 29S8 mice were infected with X31 (800 TCID50) and IFN-α (**d**), IFN-β (**e**) and IFN-λ2/3 (**f**) levels in BAL fluid were measured by ELISA (as described in methods). Data shown as the means ±s.e.m. ***P<0.0001, **P<0.001, *P<0.01 as measured by two-way ANOVA with Bonferroni post-tests.

We next wondered if the amines would show the same inhibitory effect on a mixed culture system containing various immune cell populations. We first tested CB and Histamine effects on influenza-exposed human PBMC. IFN-α/β and IFN-λ2/3 mRNA levels were significantly reduced when cells were pretreated with histamine or CB before Influenza exposure (Fig. 3a–c).

We next investigated whether amines exhibit inhibitory activity on antiviral cytokine responses *in vivo*. We determined how histamine and CB affect interferon production in 12-week-old 129S8 mice infected with the X31 Influenza strain or inoculated with vehicle control. At day 3 of influenza infection, mice pretreated with CB showed a strong reduction of IFN-α, IFN-β and IFN-λ2/3 protein production in bronchioalveolar lavage (BAL) fluid compared with untreated Influenza-infected mice (Figs 3d–f). When mice were treated with histamine before influenza infection, we noticed a trend towards interferon reduction that was not statistically significant. This result may be explained by the fact that histamine is a natural amine, and therefore degraded by histaminase found in serum^23^,^24^. Thus, CB and, to a lesser extent, histamine inhibit interferon production in Flu-stimulated complex systems such as human PBMC and *in vivo* mice infection.

### The ammonium group is essential to inhibit pDC activation

**Figure 4:**
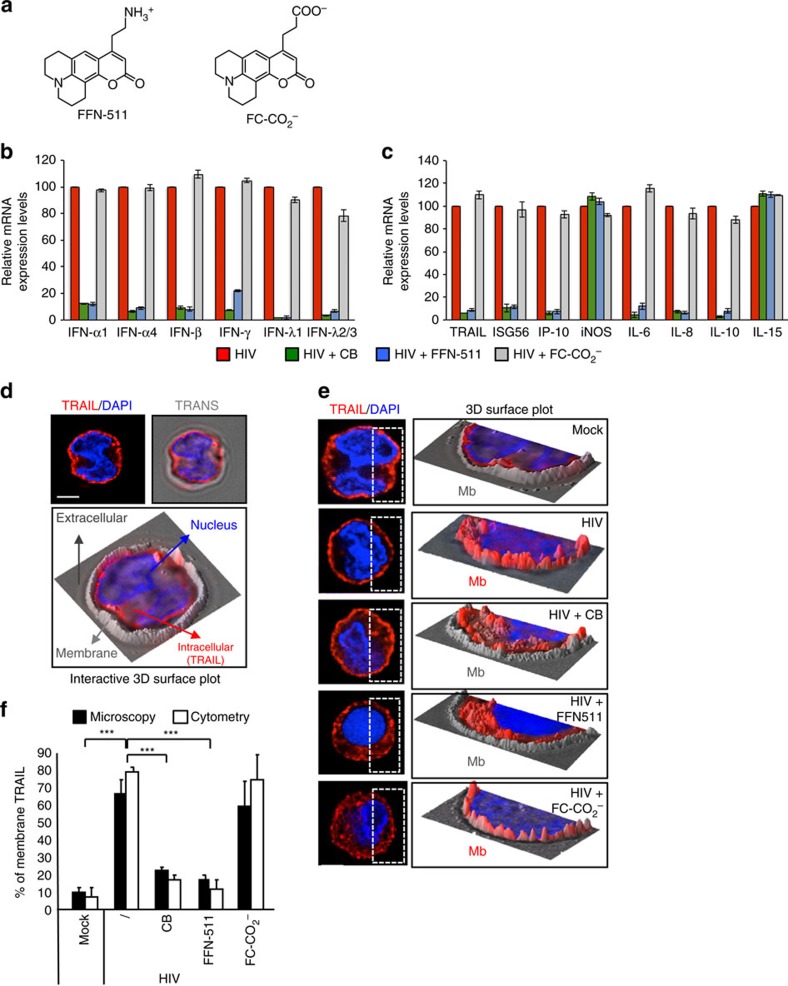
The NH_3_^+^ function is essential for inhibitory effect on pDC. (**a**) Chemical structures of FFN-511 and FC-CO_2_^−^. (**b**,**c**) mRNA levels of IFN-(α, β, γ, λ1, λ2/3) (**b**) ISG56, IP-10, iNOS and IL-(6, 8, 10, 15) (**c**) from purified pDC pre-incubated with CB, FFN-511 or FC-CO_2_^−^ (10 μM) and stimulated overnight with HIV, were measured by RT-qPCR and normalized to RPL13A. (**d**) Study of TRAIL expression by three dimensions (3D) microscopy (left panel) and 3D interactive surface plot of pDC using bright light (right panel). Cell is stained with DAPI (blue) and TRAIL (red). Plasma membrane is in grey relief. Scale bar, 3 μm. (**e**) Epifluorescence at three dimensions (3D) microscopy (left panels) of purified pDC after pre-incubation with CB, FFN-511 or FC-CO_2_^−^ (10 μM) and overnight stimulation with HIV. Cells were then stained with anti-TRAIL (red) and mounted with Fluoromount G with DAPI (blue). Scale bar, 3 μm. 3D interactive surface plot (right panels) were then acquired after ImageJ analysis. (**f**) Comparison between the 3D microscopy and flow cytometry method to quantify the percentage of membrane TRAIL of pDC after the different stimulations. Data shown as the means of three independent experiments±s.e.m. *P* values (*p*) were determined using a two-tailed Student's *t* test. ***P<0.001, **P<0.01, *P<0.05.

To further study the role of amines on pDC activation, we synthetized FFN-511 (ref. 25), a fluorescent amine mimetic of serotonin (Fig. 4a). This compound contains an ammonium group (NH_3_^+^) and a fluorescent coumarin core allowing microscopy and flow cytometry analysis. FFN-511 (50 μM), strongly reduced type I interferon production by HIV-stimulated pDC (Supplementary Fig. 2a,b) without any obvious cytotoxic effect (Supplementary Fig. 2c). To further investigate the role of the NH_3_^+^ function, we synthesized a negatively charged analogue of FFN-511, FC-CO_2_^−^, in which the ammonium group (NH_3_^+^) was replaced by a carboxylic (CO_2_^−^) moiety (Fig. 4a and Supplementary Methods). We examined the effect of the amine FFN-511 and its analogue FC-CO_2_^−^ on a panel of activation markers, using an RT-qPCR profiling assay. We selected a panel of genes that are usually activated after viral exposure: TRAIL, IFNs (IFN-α, IFN-β, IFN-γ, IFN-λ1 and IFN-λ2/3), interleukins (IL6, IL8, IL10 and IL15), chemokines (CXCL10), inducible nitric oxide synthase (iNOS) and an early ISG (ISG56). Values for each transcript were normalized to expression level of ribosomal protein L13a (RPL13A). All virus-induced genes were induced in pDC by HIV-1 and their transcription was dramatically inhibited by CB and FFN-511 but not by FC-CO_2_^−^ (Fig. 4b,c). However, neither CB nor FFN-511 affected iNOS or IL-15 gene expression (Fig. 4b,c), suggesting a specific inhibition of virus-induced gene expression rather than a global effect on cellular gene transcription.

### Amines inhibit TRAIL relocalization on HIV-stimulated pDC

We have previously shown that viral exposure results in the relocalization of TRAIL from the cytoplasm to the cell membrane^26^,^27^. Thus, we examined whether amines affect this process using three-dimensionsal (3D) microscopy^28^ (Fig. 4d). As expected, TRAIL was mostly localized in the cytosol in non-activated pDC, but became detectable at the plasma membrane on HIV-1 stimulation (Fig. 4e). CB and FFN-511 significantly inhibited TRAIL localization to the cell membrane in HIV-stimulated pDC, whereas FC-CO_2_^−^ did not. Image quantification of membrane TRAIL was performed and validated by flow cytometry results (Fig. 4f). Therefore, on HIV-1 exposure, amines prevent the surface access of an intracellular pool of TRAIL thus inhibiting the pro-killer activity of HIV-activated pDC (ref. 10).

### CXCR4 is required for amines inhibitory effect

**Figure 5:**
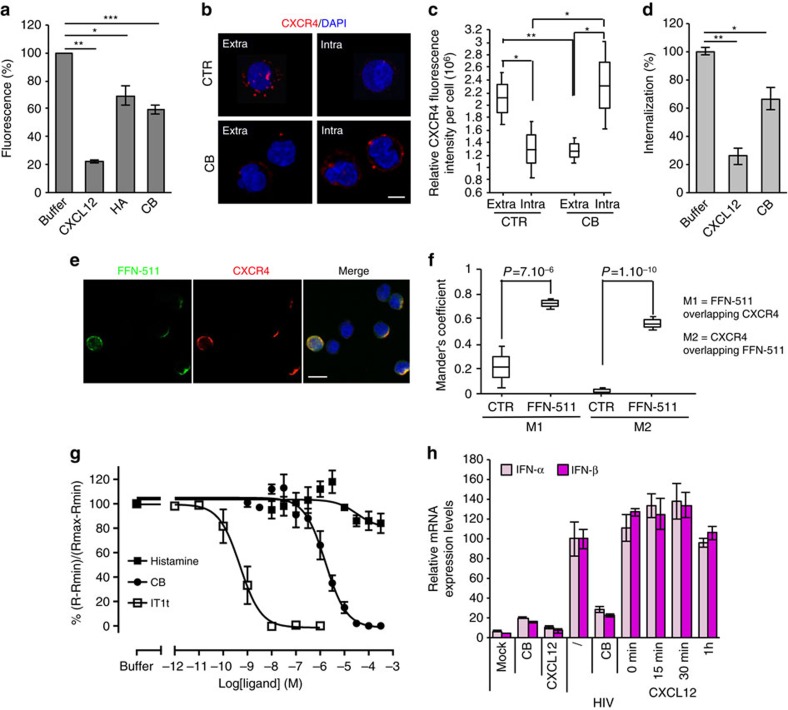
The inhibitory effect of amines on pDC requires the binding to CXCR4. (**a**) Jurkat cells were incubated with CXCL12 (100 nM), HA or CB (1 mM) at 4 °C for 30 min before staining with 12G5 antibody (anti-CXCR4) to assess compound fixation on CXCR4 by flow cytometry. (**b**) Confocal microscopy of purified pDC was performed to assess the internalization of CXCR4. Cells were incubated with 12G5 antibody at 4 °C before treatment with CB. The cells were stained with secondary antibody before or after being fixed and permeabilized, to observe extracellular or intracellular CXCR4 expression respectively. Cells were mounted with Fluoromount G with DAPI (blue). Scale bar, 7 μm. (**c**) Quantification of the intensity of fluorescence of 15 purified pDC after treatment or not. (**d**) Jurkat cells were incubated with CXCL12 (250 nM) or CB (10 μM) at 37 °C for 30 min before being stained with 1D9 antibody (anti-CXCR4) to assess internalization of CXCR4 by the compounds by flow cytometry. (**e**) Confocal microscopy of purified pDC after pre-incubation with FFN-511 (green). Cells were then stained with anti-CXCR4 (12G5) (red) and mounted with Fluoromount G with DAPI (blue). Scale bar, 12 μm. (**f**) Quantification of the colocalization between CXCR4 and FFN-511 in the cytoplasm of 20 purified pDC. Mander's Coefficient was obtained after analysis by JACoP pluging in ImageJ. M1 represents FFN-511 overlapping CXCR4 and M2 represents CXCR4 overlapping FFN-511. (**g**) Affinity of ligands for CXCR4 receptor were evaluated with the Tag-lite assay on HALOtag-CXCR4 receptors. Cells expressing HALO-tag CXCR4 were incubated with HALOtag- Lumi4-Tb (100 nM) for 1 h, then incubated in the presence of CXCL12-red tracer (5 nM) and increasing concentration of competitors. FRET signal were measured after overnight incubation at 4 °C (ref. 65). (**h**) mRNA levels of IFN-α/β from purified pDC pre-incubated with CB (10 μM) or CXCL12 (62.5 nM) for different times and stimulated overnight with HIV, were measured by RT-qPCR and normalized to RPL13A. For (c) and (f) the box represents the average±the s.d. The middle line represents the average. The whiskers represent the average±1.96 times the s.d. Data shown as the means of three independent experiments±s.e.m. *P* values (*p*) were determined using a two-tailed Student's *t* test. ***P<0.001, **P<0.01, *P<0.05.

It has been reported that compounds bearing ammonium function (NH_3_^+^) can interact with the chemokine receptor CXCR4 and induce its internalization, thus preventing CXCR4-tropic (X4) HIV-1 infection^29^. Indeed, we found that histamine and CB inhibited binding of the CXCR4 antibody 12G5 on T cells (Fig. 5a). Furthermore, we assessed intracellular and/or extracellular levels of CXCR4 by staining permeabilized and non-permeabilized pDC with receptor specific antibody. When cells were incubated with CB, most of CXCR4 was detected intracellularly, compared with untreated cells (Fig. 5b,c). Furthermore, CB-induced internalization of CXCR4 was also assessed by flow cytometry analysis of Jurkat T cells using an anti-human CXCR4 antibody clone 1D9 (ref. 30) (Fig. 5d). To visualize the interaction between CXCR4 and amines, we used the fluorescent properties of FFN-511 (ref. 25). Confocal microscopy of pDC demonstrated a strong degree of co-localization between FFN-511 and CXCR4 (Fig. 5e,f and Supplementary Fig. 3a). Competition experiments with the Tag-lite assay were performed to determine the affinity of the amines for CXCR4 (Fig. 5g) and with IT1t, a well characterized CXCR4 antagonist, as internal control^31^. Cells expressing CXCR4 fused to the HALOtag on its N-terminus were incubated 1 h with HALO-Lumi4-Tb (100 nM) and incubated at 4 °C to prevent receptor internalization in the presence of tracer, CXCL12-red (5 nM), and increasing concentrations of clobenpropit, IT1t or histamine. All the ligands inhibit CXCL12-red binding proving the ability of the ligand to bind to CXCR4. The inhibition constant for clobenpropit was 1.5+0.71 μM and as expected the IT1t inhibitory constant was subnanomolar (Ki=0.41+0.07 nM). In our test, histamine appeared to displace CXCL12-red binding at short incubation times (estimated Ki=26.5 μM at 1 h). Nevertheless, this result should be taken with caution as this effect decreases over time (Supplementary Fig. 3b,c). This progressive loss of histamine binding may be because of its degradation by histaminases present in the incubation medium^23^,^24^, or to its unspecific binding to heparin on cell membrane^32^ or to bovine serum albumin (BSA)^33^. As we have shown that our compounds could internalize CXCR4, we studied the effect of CXCR4 natural ligand, CXCL12, on pDC activation. We pre-incubated purified pDC with CXCL12 (62.5 nM) at different time points (15 min, 30 min, 60 min) before adding HIV (Fig. 5h). Whatever the time of pre-incubation, CXCL12 did not reduce IFN-α/β mRNA expressions by HIV-stimulated pDC. To confirm these surprising results we used CXCL12 at the concentration used for amines (10 μM) and even at this concentration CXCL12 did not inhibit IFN-α/β mRNA expression (Supplementary Fig. 3d). Thus, we demonstrated that CXCL12 did not act as amines and was not able to inhibit viral activation of human pDC, suggesting that these molecules might interact and activate CXCR4 in different ways.

**Figure 6:**
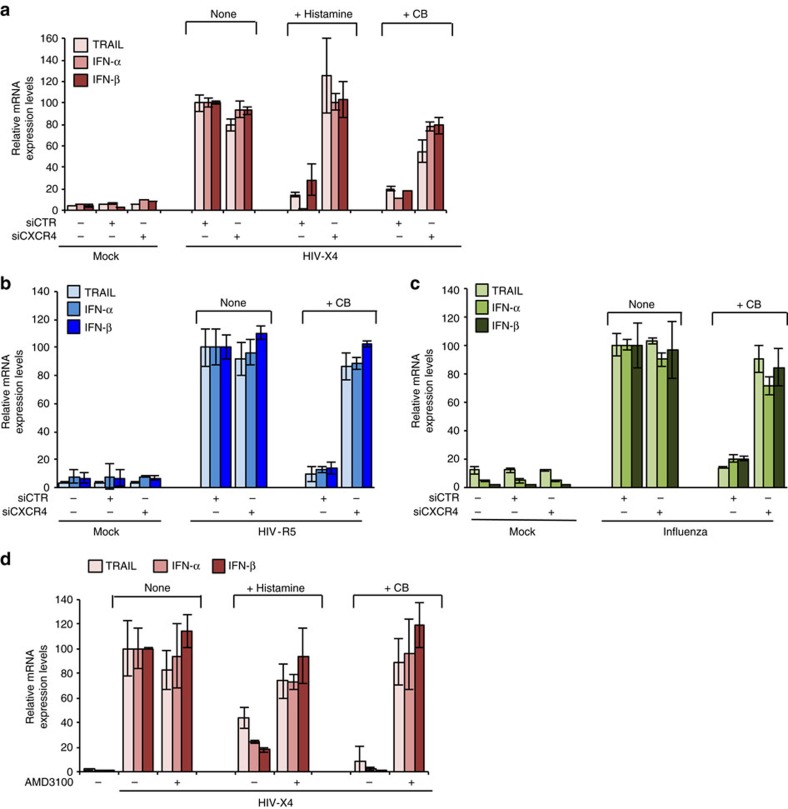
The mechanism of amines inhibitory effect on pDC requires CXCR4 expression. (**a**–**c**) pDC were treated for 24 h with a siRNA CXCR4 (siCXCR4) or a siRNA Control (siCTR) at 160 nM. Cells were pre-incubated with histamine or CB (10 μM) and then stimulated with HIV X4 (**a**), HIV R5 (**b**) or Influenza A virus (**c**). mRNA levels of TRAIL, IFN-α and IFN-β from those cells were then quantified by RT-qPCR. Data shown as the means of three independent experiments ±s.e.m. (**d**) mRNA levels of TRAIL, IFN-α and IFN-β from purified pDC pre-incubated with AMD-3100 (20 μM) then histamine or CB (10 μM) and stimulated overnight with HIV-X4, were measured by RT-qPCR and normalized to RPL13A. Data shown as the means of three independent experiments±s.e.m. *P* values (*p*) were determined using a two-tailed Student's *t* test. ***P<0.001, **P<0.01, *P<0.05.

We then silenced CXCR4 in pDC using small interfering RNA (siRNA) (Supplementary Fig. 4a,b). CXCR4 gene silencing suppressed the inhibitory effect of histamine or CB on type I interferon and TRAIL, in pDC stimulated by CXCR4-tropic HIV-1 (Fig. 6a). It should be noticed that CXCR4 is not required for pDC activation by HIV-1 (refs 22, 34). To generalize our findings, we verified that CXCR4 silencing also blocked CB inhibitory effect on pDC activated by a CCR5-tropic (R5) HIV-1 and Influenza (Fig. 6b,c). Thus, amines inhibit virus sensing in pDC by engagement of CXCR4.

We then evaluated whether the well-known CXCR4 antagonist AMD3100 (ref. 35) could inhibit interferon production on HIV-stimulated pDC. Interestingly, we confirmed that AMD3100 alone did not block IFN-α/β nor TRAIL expression by HIV-activated pDC (refs 22, 34) (Fig. 6d), suggesting a different mechanism of action than the amines. We then tested whether AMD3100 is able to block amine action by limiting the access. We also quantified the effect of the amines on pDC activation treated or not with AMD3100. Purified cells were pre-incubated with AMD3100 for 1 h and then followed by histamine or CB for 1 h and finally exposed to HIV-1 overnight. AMD3100 drastically abolished biological activities of histamine and CB on HIV activated-pDC. Indeed, AMD3100 treatment restored IFN-α/β mRNA and protein, and TRAIL productions in the presence of histamine or CB in HIV-activated pDC (Fig. 6d; Supplementary Fig. 5a). These results were confirmed on a panel of pDC cytokine secretion (IFN-γ and IL6) and ISG (ISG56) (Supplementary Fig. 5b–d). Altogether, these results unambiguously demonstrate that CXCR4 is required for the inhibitory activity of amines on pDC activation and that the amines interact with CXCR4 through a mechanism of action different from CXCL12 and AMD3100.

## Discussion

Plasmacytoid dendritic cells are innate immune cells implicated in multiple diseases because of their capacity of secreting massive levels of type I interferon, in particular IFN-α (ref. 36). This cytokine provides essential immunity against viruses, and critically contributes to the reduction of viral spread. However, since the over production of type I interferon may also have deleterious effects, pDC response should be under tight control^37^.

Natural amines, which are present in all eukaryotic cells, display multifunctional characteristics during cell differentiation and exhibit immunomodulatory functions^38^,^39^. Previous studies described histamine as a strong inhibitor of type I interferon and cytokine production by pDC through histamine receptors (H4R)^19^,^20^. Using histamine and the selective H4R agonist, clobenpropit (CB), we showed that these two molecules strongly inhibit type I interferon production by HIV-1, Influenza-A or dengue virus-activated pDC. However, the use of an H4R antagonist and of H4R KO murine cells did not impact CB effect on IFN-α production by activated pDC. This unexpected result established that the effect of CB is independent of histamine receptors. We further show that histamine and histamine analogues (CB) were not the only compounds with immunomodulatory function on pDC, as other natural aromatic amines (serotonin and dopamine) also inhibited type I interferon production and membrane TRAIL expression by HIV-activated pDC. We next extended our study to mixed cultures, which confirmed that histamine and CB can exert inhibitory activity in the context of multiple immune cell types. To extend our findings on the inhibitory potential of amines *in vivo*, we tested the natural amine histamine and the synthetic CB in a mouse model, in which induction of type I and type III interferon production by Influenza was well characterized^16^. Mice pretreated with CB showed a massive reduction of types I and III interferon productions, validating that amines have inhibitory activity *in vivo*. Histamine treatment led to only partial reduction of interferon production. However, micromolar concentration of histamine is required to block interferon production *in vitro* in virus-activated pDC and this concentration could only be raised physiologically in local tissues when professional cells like mastocytes are activated and only temporarily before it is rapidly degraded by histaminases in plasma^23^,^40^. The absence of *in vivo* effect of histamine could therefore be explained by an insufficient dose and a shorter half-life of this natural molecule. Our results suggest that modulation of interferon by amine-like small molecule drugs may represent a novel therapeutic approach in situations where excessive interferon drives inflammation, autoimmunity or immunopathology.

Natural (histamine, dopamine and serotonin) and synthetic (CB) molecules do not exhibit any evident structural similarities besides the presence of a NH_3_^+^ and NH_2_^+^ cation at physiological pH. We synthetized the well characterized fluorescent serotonin analogue, FFN-511 (ref. 25) to better understand the role of the amino group on pDC modulation. FFN-511 inhibited the expression of several cytokines, including types I, II, III interferons, and activation of various ISG, in HIV-activated pDC. We next validated the central role of the NH_3_^+^ group by synthetizing an FFN-511 strict fluorescent analogue (FC-CO_2_^−^), in which the ammonium (NH_3_^+^) was replaced by a carboxylate function (CO_2_^−^). In contrast to FFN-511, FC-CO_2_^−^ did not show any inhibitory effect on pDC activation, highlighting an essential role for the NH_3_^+^ function in the immunomodulatory activity of amines.

These monoamines and polyamines exhibited similar inhibitory effects on pDC, suggesting that they acted *via* one common receptor. Interestingly, polyamine derivatives prevent infection by CXCR4-tropic but not CCR5-tropic HIV-1 strains, suggesting an interaction with the chemokine receptor CXCR4 (ref. 41). Furthermore, it has been shown that development of mouse and human plasmacytoid dendritic cells in bone marrow required CXCL12-CXCR4 chemokine signalling^42^,^43^. We evaluated the potential interaction between CXCR4 and amines. We report that CB induced CXCR4 internalization in pDC, and that FFN-511 strongly colocalized with CXCR4. Furthermore, CXCR4 silencing reverted CB inhibitory effect on pDC activated with Influenza or with X4 or R5 HIV-1. Altogether, our results demonstrated that CXCR4 is required by the amines to inhibit viral activation of pDC.

We showed that amine's activity is abrogated by the use of CXCR4 siRNA, demonstrating that amines-mediated inhibition of pDC required CXCR4. To block the access of the amines to CXCR4, we used the well-described CXCR4 antagonist, AMD3100. We then showed that AMD3100 by itself had no effect on pDC but drastically reduced the inhibitory effect of CB on IFN-α/β and TRAIL mRNA expression by HIV-activated pDC. Furthermore, it seems that internalization of CXCR4 is an important step in the inhibition of pDC activation. However, pDC activation was not affected by the natural ligand CXCL12, even at high concentrations, while CXCL12 induces G protein and β-arrestin activation of CXCR4 and therefore its internalization^44^,^45^. Thus, CXCR4 internalization seems required for the inhibitory effect of amines but is not sufficient. Altogether, these observations suggest that amines do not act as ‘classical' ligands or antagonists of CXCR4. Further investigations will be required to unravel the precise molecular mechanism that connects amine-induced CXCR4 downstream signalling of TLR7-mediated pDC activation as well as mutagenesis study to precisely characterize CXCR4 amino compounds binding pocket.

Our study provides new insights into the regulation of pDC activity by endogenous molecules, opening new perspectives for the development of therapeutic strategies. Indeed, pDC activation might be a double-edge sword for the host depending on the disease context, and its regulation represents a strategic issue in chronic infections and autoimmune diseases in which pDC are suspected to be hyperactive. For instance, increased IFN-α levels in the serum of Systemic Lupus Erythematous (SLE) patients correlate with both, disease activity and the expression of prognosis markers^46^. Pathologic over-activation of pDC and massive type I interferon production also leads to increased apoptotic ligand expression such as TRAIL. Inhibition of type I interferon-induced TRAIL expression may reduce symptoms in cutaneous lupus erythematous by decreasing autoantibody production and autoimmune tissue injury^47^. Indeed, pDC depletion showed a positive clinical impact in a mouse lupus model^48^. TRAIL-dependent immune suppression during sepsis is deleterious and TRAIL neutralization might be a potential therapeutic target in septic patients^49^. In addition, prolonged type I interferon production by pDCs could also have damaging consequences during infections. Two recent studies have illustrated this deleterious effect, demonstrating that type I interferon facilitate persistent infection and disease progression in mice infected with lymphocytic choriomeningitis virus (LCMV)^50^,^51^. Indeed, amine-mediated type I interferon regulation has strong therapeutic potential in a variety of clinical situations where excessive amounts of this cytokine promote disease. These situations range from autoimmune diseases such as Lupus, which were shown to be driven by type I interferon^13^, via acute and chronic viral infections to various bacterial infections, where type I interferon were shown to suppress antibacterial immune responses^52^. Furthermore, viral-bacterial co-infections are a common complication of many viral infections including influenza, and one central mechanism of the facilitated bacterial outgrowth is type I interferon-mediated block of antibacterial responses^52^. Lastly, the growing number of Mendelian disorders associated with excessive type I interferon and known as interferonopathies, such as the Aicardi-Goutieres syndrome^53^, could be treated with small molecules reducing type I interferon production.

The role of type I interferon and pDC during HIV-1 infection remains complex^54^. pDC largely contribute to the reduction of viral spread by producing important levels of IFNs^55^. However, the role of type I interferon in HIV-1 may depend on the stage of the disease^56^. Although type I interferon have potent antiviral and immune stimulatory effect during acute infections, they could also have deleterious consequences in the long term^57^. During the course HIV-1 infection, sustained IFN signalling contributes to chronic immune activation and pathogenesis^58^,^59^. Interestingly pDC from women produce significantly higher levels of IFN-α in response to HIV-1 than pDC from men, and that these higher levels of IFN-α lead to faster progression to AIDS^60^. Furthermore, Stary *et al* showed that TRAIL-expressing killer pDC are implicated in apoptosis of CD4^+^ T cells in tonsils from HIV-infected progressor patients^11^. Thus, blocking type I interferon during the chronic phase of HIV-1 infection may be beneficial^61^.

In summary, our study establishes an essential link between natural amines and inhibition of the innate immune system. We demonstrate here that natural amines are efficient inhibitors of viral activation of pDC through CXCR4 engagement. We clearly identify CXCR4 as a potential ‘on/off' switch of pDC activity and placed this receptor as a new therapeutic target. Various pharmacological agents targeting CXCR4 are currently under clinical investigation^62^, and could provide ‘ready-to-use' drugs to down-modulate pDC function and reduce type I interferon levels. Compared with mAb-mediated type I interferon-blockade, this small-molecule approach may rapidly lead to economically viable treatments for a variety of unmet medical needs associated with excessive type I interferon production.

## Methods

### Blood samples isolation and culture of blood leucocytes

Blood from healthy HIV-1-seronegative blood bank donors was obtained from ‘*Etablissement Français du Sang*' (convention # 07/CABANEL/106), Paris, France. Experimental procedures with human blood have been approved by Necker Hospital Ethical Committees for human research and were done according to the European Union guidelines and the Declaration of Helsinki and informed consent was obtained from all donors. *In vitro* experiments were performed using human peripheral blood mononuclear cells (PBMC) isolated by density centrifugation from peripheral blood leucocyte separation medium (Cambrex, Gaithersburg, MD). Human pDC were purified by negative selection with the Human plasmacytoid DC enrichment kit (StemCell Technologies). Cells were cultured in RPMI 1640 (Invitrogen, Gaithersburg, MD) containing 10% fetal bovine serum (Hyclone, Logan, UT). After purification, we obtained purity higher than 91% for pDC.

### Viral stimulation and infection

PBMC were seeded at 1.10^6^ per 1 ml or purified pDC were seeded at 5.10^4^ per 100 μl and then stimulated with the following viruses: inactivated AT-2 HIV-1_MN_ (CXCR4 co-receptor specific) or AT-2 HIV-1_ADA_ (CCR5 co-receptor specific) at 60 ng ml^−1^ p24^CA^ equivalent (kindly provided by J.D. Lifson (SAIC-NCI, Frederick, MD)), Infectious human Influenza A/PR/8/34 virus (Flu), titre 1:8192 at dilution 1:1,000 or DENV-2 16681 at MOI 10. Infectious HIV-1_MN_ (tissue culture 50% infective dose (TCID50)=106) and HIV-1_ADA_ (TCID50=1,000) were used at the same concentration. Purified pDC were pre-treated with amino compounds for 1 h, following overnight stimulation with virus. Supernatants were collected for cytokine detection. Microvesicles isolated from uninfected cell cultures matched to the culture to produce the virus were used as negative control (Mock).

### Chemical compounds

Histamine dihydrochloride, clobenpropit dihydrobromide, dopamine and serotonin (Sigma-Aldrich, MO,USA) were diluted in pure water. The compounds were added in pDC culture at 10 μM (or other if specified) 1 h before stimulation or not with the different viruses. For histamine, *X-vivo* culture media (Lonza) was used in order to avoid histaminases. Fluorescent compounds FFN-511 and FC-CO_2_^−^ were synthetized as described in supplementary material. When stated, AMD (20 μM) (Sigma-Aldrich, MO, USA) was added to the cells 1 h before incubation with. The oligodinucleotide A151 (TTAGGG)_4_ ODN (Integrated DNA Technologies, Coralville, IA) was used at 5 mM. The histamine receptors antagonists (pyrilamine/PYR for H1R, cimetidine/CIM for H2R, thioperamide/THIO for H3R and JNJ7777120/JNJ and A943931 for H4R) (Sigma-Aldrich, MO,USA) were used at 10 μM.

### H4R and CXCR4 knockout experiments

pDCs were seeded at 10^5^ cells per 100 μl in 96-well plates and incubated at 37 °C. The experiments were performed using protocol as previously described by Smith *et al*.^63^. Briefly, H4R and CXCR4 Small interfering RNA (siRNA) (ON-TARGET SmartPool, Dharmarcon) were diluted in PBS and added to an equal volume of DOTAP (Roche Applied Sciences). The mix was gently mixed and incubated at room temperature during 15 min. After incubation, the mix was added to cells in culture at a final concentration of 160 nM. Finally, cells were incubated at 37 °C for 24 h before adding the different stimulations overnight. Control was performed using a siRNA control (ON-TARGET SmartPool, Dharmarcon).

### Flow Cytometry

Cultured cells were incubated for 20 min at 4 °C with the following antibodies mouse IgG1 PE-conjugated TRAIL clone RIK-2 (1/100) (BD Bioscience, San Jose, CA), mouse IgG1 APC-conjugated BDCA-4 clone REA380 (1/100), mouse IgG2a FITC-CD123 clone AC145 (1/100) (Miltenyi, Bergisch Gladbach, Germany), mouse IgG2a FITC-HLADR clone L243 (1/200), mouse IgG2a PercP-cy5.5-CCR7 clone G043H7 (1/50), mouse IgG1 APC-CD40 clone 5C3 (1/50), mouse IgG1 BV421-CD80 clone 2D10 (1/50), mouse IgG2b AF488-CD86 clone IT2.2 (1/50), mouse IgG1 PE-CXCR4 clone 12G5 (1/100) (Biolegend, San Diego, CA) or with appropriate isotype-matched control antibodies (5 μg ml^−1^ each) in PBS containing 2% foetal bovine serum (Sigma, Saint Louis, MO) and FC-receptor blockers (BD Biosciences, San Jose, CA). Flow cytometry analysis was performed on a BD Canto II or LSR II flow cytometer using flow cytometry Diva software (BD Biosciences, San Jose, CA). FlowJo software (Treestar, Ashland, OR) was used to analyse data.

### Cytokine detection

pDC supernatants were tested for IFN-α by ELISA using the Human IFN Alpha Multi-Subtype ELISA Kit (TCM) from PBL Assay Science, NJ, USA, catalogue number 41105-1 according to the manufacturer's instructions.

### RT-qPCR analyses

Total RNA was extracted using RNeasy Micro kit and was submitted to DNase treatment (Qiagen), following manufacturer's instructions. RNA concentration and purity were evaluated by spectrophotometry (Biophotometer, Eppendorf). RNA of 500 ng was reverse-transcribed using PrimeScript RT Reagent Kit (Perfect Real Time, Takara) in a 10 μl reaction. Real-time PCR reactions were performed in duplicates using Takyon ROX SYBR MasterMix blue dTTP (Eurogentec) on a 7900HT Fast Real-Time PCR System (Applied Biosystems). Transcripts were quantified using the following program: 3 min at 95 °C followed by 35 cycles of 15 s at 95 °C, 20 s at 60 °C and 20 s at 72 °C. Values for each transcript were normalized to expression levels of RPL13A (60 S ribosomal protein L13a) using the 2−ΔΔCt method. Primers used for quantification of transcripts by real time quantitative PCR are the following:

RPL13A: forward primer, 5′-CCTGGAGGAGAAGAGGAAAGAG-3′; reverse primer, 5′-TTGAGGACCTCTGTGTATTTGTCAA-3′

TRAIL: forward primer, 5′-GCTGAAGCAGATGCAGGACAA-3′; reverse primer, 5′-TGACGGAGTTGCCACTTGACT-3′

IFN-α1/13: forward primer, 5′-CCAGTTCCAGAAGGCTCCAG-3′; reverse primer, 5′-TCCTCCTGCATCACACAGGC-3′

IFN-α4/10: forward primer, 5′-CCCACAGCCTGGGTAATAGGA-3′; reverse primer, 5′-CAGCAGATGAGTCCTCTGTGC-3′

IFN-β: forward primer, 5′-TGCATTACCTGAAGGCCAAGG-3′; reverse primer, 5′-AGCAATTGTCCAGTCCCAGTG-3′

IFN-λ1: forward primer, 5′-GGACGCCTTGGAAGAGTCAC-3′; reverse primer, 5′-CTGGTCTAGGACGTCCTCCA-3′

IFN-λ2/3: forward primer, 5′-GGGCCTGTATCCAGCCTCAG-3′; reverse primer, 5′-GAGGAGGCGGAAGAGGTTGA-3′

IFN-γ: forward primer, 5′-GGCAGCCAACCTAAGCAAGAT-3′; reverse primer, 5′-CAGGGTCACCTGACACATTCA-3′

IL6: forward primer, 5′-TAACCACCCCTGACCCAACC-3′; reverse primer, 5′-ATTTGCCGAAGAGCCCTCAG-3′

IL8: forward primer, 5′ -AAGGGCCAAGAGAATATCCGAA-3′; reverse primer, 5′-ACTAGGGTTGCCAGATTTAACA-3′

IL10: forward primer, 5′-GGGCTTGGGGCTTCCTAACT-3′; reverse primer, 5′-GCTGGCCACAGCTTTCAAGA-3′

IL15: forward primer, 5′-AAGAAGAGCTGGCTATGGCA-3′; reverse primer, 5′-TCATGTTCCATGCTGCTGAC-3′

ISG56: forward primer, 5′-AGGACAGGAAGCTGAAGGAG-3′; reverse primer, 5′-AGTGGGTGTTTCCTGCAAGG-3′

CXCL10: forward primer, 5′-CGCTGTACCTGCATCAGCAT-3′; reverse primer, 5′-GCAATGATCTCAACACGTGGAC-3′

iNOS: forward primer, 5′-CAGCGGGATGACTTTCCAA-3′; reverse primer, 5′-AGGCAAGATTTGGACCTGCA-3′

CXCR4: forward primer, 5′-GCATGACGGACAAGTACAGGCT-3′; reverse primer, 5′-AAAGTACCAGTTTGCCACGGC-3′

H4R: forward primer, 5′-ACACGCTGTTCGAATGGGAT-3′; reverse primer, 5′-TCGATCATAGCTGATGAGGACAA-3′.

### *In vivo* treatment of mice

Male mice 129S8 of 12 week old (Jackson Laboratory), bred at the MRC-National Institute for Medical Research (NIMR) under specific pathogen–free conditions as previously described^16^, were treated with Clobenpropit dihydrobromide (Sigma-Aldrich, C209) (450 μg per 30 μl per mouse), Histamine dihydrochloride (Sigma-Aldrich, H7250) (450 μg per 30 μl per mouse) or Vehicle Control (PBS) (30 μl per mouse) 18 h before infection. Mice were infected with Influenza A virus strain X31 (H3N2) (a kind gift from Dr J. Skehel, MRC-NIMR) at 800 TCID_50_/30 μl. X31 was grown in the allantoic cavity of 10 day-embryonated hen's eggs and was free of bacterial, mycoplasma, and endotoxin contamination, stored at −70 °C and titrated on MDCK cells, according to the Spearman-Karber method. All mice were treated and infected intranasally (i.n) under light isoflurane-induced anaesthesia. At 3 days post infection mice were killed and bronchioalveolar lavage (BAL) fluid was collected. BAL samples were centrifuged at 1,300 r.p.m., 5 min at 4 °C and supernatant collected. Samples were then analysed for concentrations of IFNα, (eBioscience) IFNβ (Biolegend UK) and IFNλ (R&D) by ELISA as per the manufacturer's instructions. All protocols for breeding and experiments with animals were approved by the local ethical committee of the MRC-NIMR and are part of a project licence approved by the Home Office, UK, under the Animals (Scientific Procedures) Act 1986.

### Three dimensional microscopy

In some cases, purified pDC cells cultured overnight in presence of HIV-1 and with the different compounds (CB, FFN-511 and FC-CO_2_^−^). pDC (1 × 10^5^ cells per slide) were plated on collagen-coated slides and fixed with paraformaldehyde 4%. Cells were then incubated with mouse IgG1 anti-CXCR4 antibody clone 12G5 (1/50) (Biolegend, San Diego, CA) in saturation buffer PBS-BSA 0,5% for membrane staining or in permeabilizing buffer containing 1% saponin with monoclonal mouse IgG1 anti-TRAIL clone RIK2 (1/50) (Biolegend, San Diego, CA, USA). CXCR4 was revealed by a donkey anti-mouse IgG-AF647 (1/400) (Molecular Probes, OR, USA) and TRAIL was revealed by a Donkey anti-mouse IgG-Cyanine 3 (1/200) (Jackson ImmunoResearch, West Grove, PA). Nucleus was stained using DAPI (Molecular Probes, Paisley, UK). Slides were mounted with Fluoromount-G (eBioscience, CA, USA) and scanned with a Nikon Eclipse 90i Upright microscope (Nikon Instruments Europe, Badhoevedorp, The Netherlands) using a × 100 Plan Apo VC piezo objective (NA 1.4) and Chroma bloc filters (ET-DAPI, ET-GFP). Images were subsequently deconvoluted with a Meinel algorithm and 8 iterations and analysed using Metamorph (MDS Analytical Technologies, Winnersh, UK). TRAIL/DAPI/Overlay/Confocal plane: Representative 2D focal plan. Overlay with bright: Bright. Reconvolution overlays: 2D projections of the maximum intensity pixels along the *Z*-axis.

In other cases, cells were cultured overnight in media alone then were washed in ice-cold PBS-BSA 0,2% and stained with CXCR4 antibody (R&D Systems) for 1 h at 4 °C. Cells were then washed with PBS-BSA 0,2% and stimulated with CB for 1 hour at 4 °C. Data are expressed as the mean percentage±s.e.m. mean channel fluorescence intensity (MFI) values for residual surface expression and intracellular staining. After staining in suspension, pDC (1 × 10^5^ cells per slide) were spun for 10 min at 400 r.p.m. with a Shandon Cytospin Cytocentrifuge (Thermo Scientific, St-Herblain, France) and fixed with paraformaldehyde 4%. Cells were then stained with secondary antibody anti-mouse-AF647 (Molecular Probes, OR, USA) either at the membrane or after permeabilization with Triton 0.2%. Finally, slides were washed in PBS, counterstained with Hoechst 33342 and mounted in Fluoromount-G medium. Images were digitally acquired with a Zeiss LSM 710 confocal Microscope using 63 × PL APO O.N.=1.4 per oil objective.

All analysis was performed using the ImageJ software (NIH, Bethesda, MD, USA).

### Image Quantification

Seven pictures were taken from each slide for each Z section framing the nucleus. Quantification of the co-localization in the cytoplasm of purified pDC using Mander's Coefficient was obtained after analysis by JACoP pluging in ImageJ.

### CXCR4 internalization

#### Interaction with CXCR4

CB and histamine binding to CXCR4 was assessed by flow cytometry analysis (FACSCantoII; Becton Dickinson) of Jurkat cells (ATCC) using anti-human CXCR4 antibodies^30^. Briefly, Jurkat cells were pre-incubated with CB, histamine (1,000 μM) or buffer for 30 min at 4 °C in FACS buffer (PBS-1% FCS). After incubation, cells were washed with FACS buffer by centrifugation, then stained with mouse IgG1 PE-labelled anti-human CXCR4 antibodies 12G5 (Pharmingen) for 30 min at 4 °C. After being washed, the cells were fixed with 4% paraformaldehyde in FACS buffer for 5 min at 4 °C. CXCR4 staining was quantified by flow cytometric analysis (10,000 cells per sample) on a cytometer (FACSCantoII, Becton-Dickinson). Data were processed using FACSDiva software (Becton Dickinson). All values represent mean fluorescence intensities of cells relative to CXCR4 levels in buffer-treated cells (100%) from a triplicate experiment±s.d. Statistical calculations were performed with a two-tailed paired Student's *t*-test using GraphPad Prism Version 5.03. *P*<0.05 was considered significant.

#### Internalization of CXCR4

Internalization of CXCR4 was assessed by flow cytometry analysis of Jurkat cells using an anti-human CXCR4 antibody^30^. Briefly, Jurkat cells were pre-incubated with CB (10 μM), CXCL12 (250 nM) or buffer for 30 min at 37 °C in serum-free medium. After incubation, cells were washed with FACS buffer by centrifugation, then sequentially stained with rat PE-labelled anti-human CXCR4 antibody clone 1D9 (BD Pharmingen) for 30 min at 4 °C. After being washed, the cells were fixed with 4% paraformaldehyde in FACS buffer for 5 min at 4 °C. CXCR4 expression was quantified by flow cytometric analysis (10,000 cells per sample) on a cytometer. Data were processed using FACSDiva software (Becton Dickinson). All values represent mean fluorescence intensities of cells relative to CXCR4 expression in buffer-treated cells (100%) from a triplicate experiment±s.d. Statistical calculations were performed with a two-tailed paired Student's *t*-test using GraphPad Prism Version 5.03. *P*<0.05 was considered significant.

### Binding assays

CHO cells were plated in black-walled, dark-bottom, 96-well plates (Greiner CELLSTAR plate; Sigma-Aldrich, St Louis, MO, USA) at 30,000 cells per well in DMEM containing 10% Fetal Calf serum (Lonza), and 1% non-essential amino acids (GIBCO) and transfected 24 h later with a plasmid coding for CXCR4 fused at its N-terminus to the HALOTag (HALO-CXCR4). Briefly, a mix of isotonic NaCl solution (50 μl per well) containing JetPei (0.6 μl), SNAP-CXCR4 coding plasmid (10 ng) and non-coding plasmid (190 ng) were added to the culture medium (100 μl). Cells were labelled as previously described^64^. Briefly, the day after the transfection, cells were rinsed one with Tag-lite medium (Cisbio Bioassays, Codolet, France) and incubate in the presence of Tag-lite medium containing 100 nM HALOTag-Lumi4-Tb for at least one hour. Cells were then washed four times and incubated in the presence of CXCL12-red (5–10 nM) and increasing concentration of competitors. Fluorescent signal was measured at 620 nm (fluorescence of the donor) and at 665 nM (FRET signal) over 1 h on a Pherastar (BMG LABTECH, Champigny s/Marne, France). Results were expressed as the 665/620 ratio. Specific variation of the 665/620 ratio was plotted as a function of competitor concentration. All binding data were analysed with Prism 6 (GraphPad Software, Inc., San Diego, CA) using the one site-specific binding equation. All results are expressed as the mean±s.e.m. of at least three independent experiments. For all competitions, three independent experiments were performed in triplicate. Kis were calculated from IC50 values with the Cheng Prusoff equation^65^. The affinity of CXCL12-red is equal to 48 nM (ref. 66).

### Statistical analysis

For cell data, data shown as the means±s.e.m. Sample sizes for this exploratory study were designed to ensure statistical results, while experimental feasibility and minimization of donors. *P* values (*P*) were determined using a two-tailed Student's *t*-test. *P*<0.05 was considered statistically significant. *P<0.05; **P<0.01 and ***P<0.001. GraphPad Prism five was used to confirm equal variances in between groups. Univariate distributions of flow cytometry data were performed by probability binning, in 300 bins using FlowJo software.

For mice data, data shown as the means±s.e.m. Sample sizes for mice data were designed to give statistical power, not randomized and not blinding, while minimizing animal use. Data sets were analysed by two-way ANOVA with Bonferroni posttests (cytokine concentration time courses). GraphPad Prism 5 (GraphPad Software, San Diego, CA) was used for data analysis and preparation of all graphs. *P* values <0.01 were considered to be statistically significant.

### Data availability

The data that support the findings of this study are available from the corresponding author on request.

## Additional information

**How to cite this article**: Smith, N. *et al*. Natural amines inhibit activation of human plasmacytoid dendritic cells through CXCR4 engagement. *Nat. Commun.* **8**, 14253 doi: 10.1038/ncomms14253 (2017).

**Publisher's note**: Springer Nature remains neutral with regard to jurisdictional claims in published maps and institutional affiliations.

## Supplementary Material

Supplementary Information Supplementary Figures, Supplementary Methods and Supplementary References

## Figures and Tables

**Table 1:** Summary of the efficiency and therapeutic index of the monoamines.

**Compounds**	**EC50 (μM)**	**TC50 (μM)**	**Therapeutic index**
Histamine	1.4±1	<2 mM	<1,500
Clobenpropit	24±2	417±6	17.4
Serotonin	1.3±0.5/113±3	1500±54	1,150/13.3
