# Supplementary material for: Natural amines inhibit activation of human plasmacytoid dendritic cells through CXCR4 engagement
Source: Nat Commun. 2017 Feb 9;8:14253. doi: 10.1038/ncomms14253 (PMC5309800; doi:10.1038/ncomms14253)
Supplement: Supplementary Information — Supplementary Figures, Supplementary Methods and Supplementary References [file ncomms14253-s1.pdf]

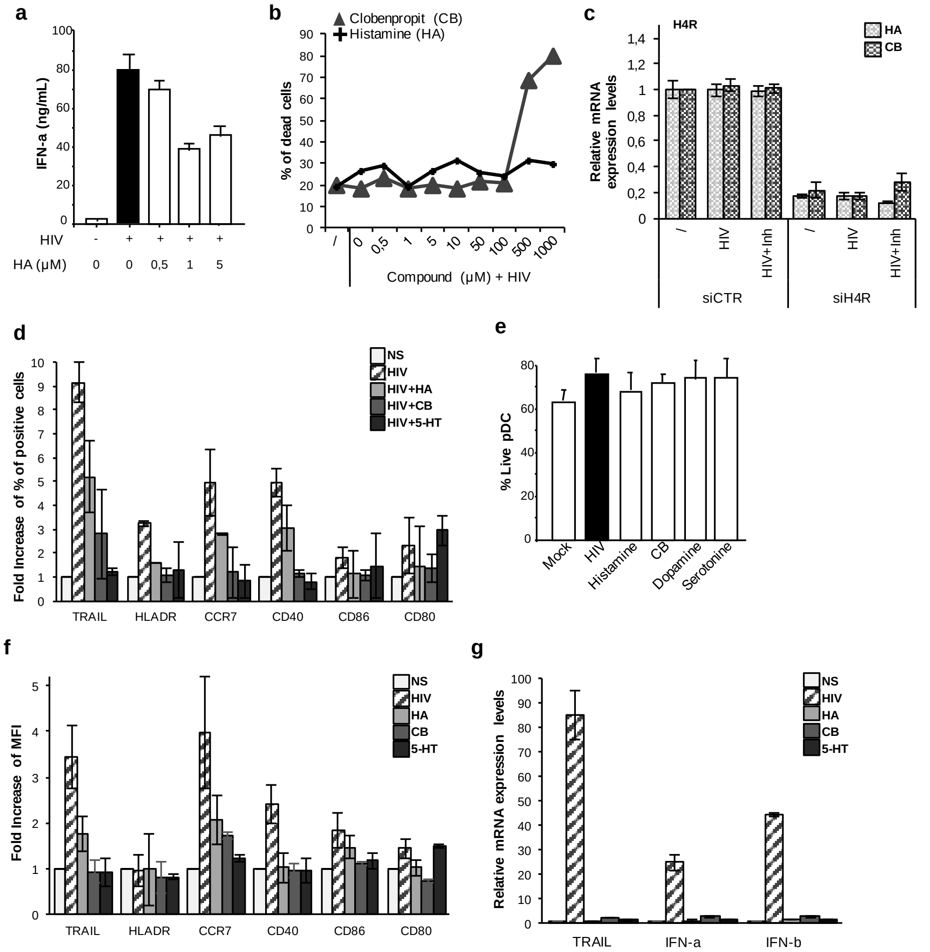

Supplementary Figure 1. Natural amines inhibit pDC activation and are not toxic to pDC. (a) Isolated pDC were pretreated with histamine at the various concentrations and then stimulated with microvesicles (mock) alone or with HIV overnight. IFN-α was measured in the supernatants by Elisa. **(b)** Viability assessed by the Live-Dead method by flow cytometry after 24h on PBMC in culture with histamine and CB at the various concentrations. **(c)** pDC were treated for 24h with a siRNA H4R (siH4R) or a siRNA Control (siCTR) at 160nM. Cells were pre-incubated with HA or CB (10µM) and then stimulated with HIV X4. mRNA levels of H4R from those cells were then quantified by RT-qPCR. **(e)** Viability assessed by the Blue Trypan method after 24h on PBMC in culture with histamine, CB, dopamine and serotonine at the concentration of 10µM. **(d)** and **(f)** Level of membrane TRAIL, HLADR, CCR7, CD40, CD86, CD80 was assessed by flow cytometry on purified pDC after pre-incubation with histamine, CB and serotonin with overnight HIV stimulation **(d)** or compounds alone **(f)**. **(g)** mRNA levels of TRAIL, IFN-α and IFN-β from purified pDC pre-incubated with HIV, histamine, CB and serotonin were measured by RT-qPCR and normalized to RPL13A. Error bars indicate the mean and SEM. Data shown are representative of three independent experiments.

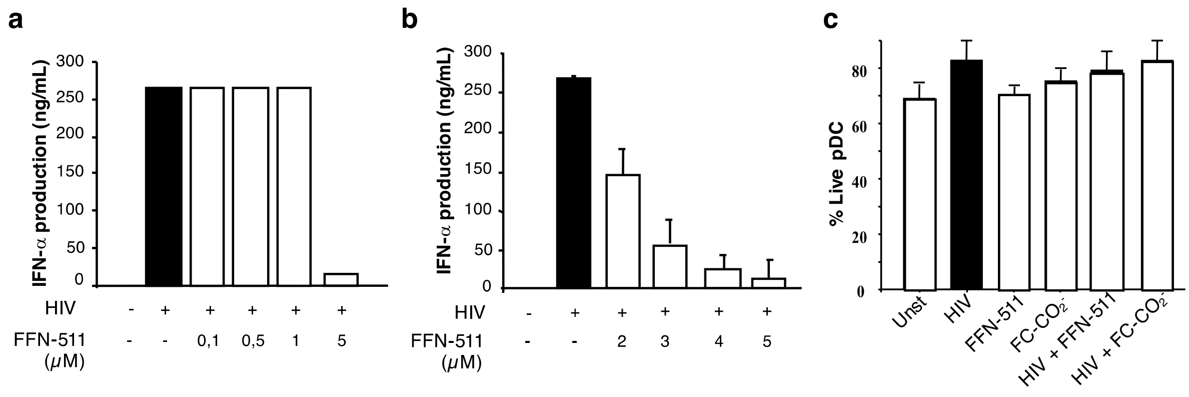

Supplementary Figure 2. FFN-511 inhibits pDC activation and the synthetic compounds are not toxic to pDC. (a) IFN-α was measured in supernatants of isolated pDC pretreated with FFN-511 at the different concentrations and then stimulated with HIV overnight. **(b)** IFN-α was quantified in supernatants of purified pDC after pre-incubation with FFN-511 at various concentrations and then stimulation overnight with HIV. **(c)** Viability assessed by the trypan blue method of pDC in culture with FFN-511 and FC-CO2- at the concentrations of 50μM stimulated or not overnight with HIV. Error bars indicate the mean and SEM. Data shown are representative of three independent experiments.

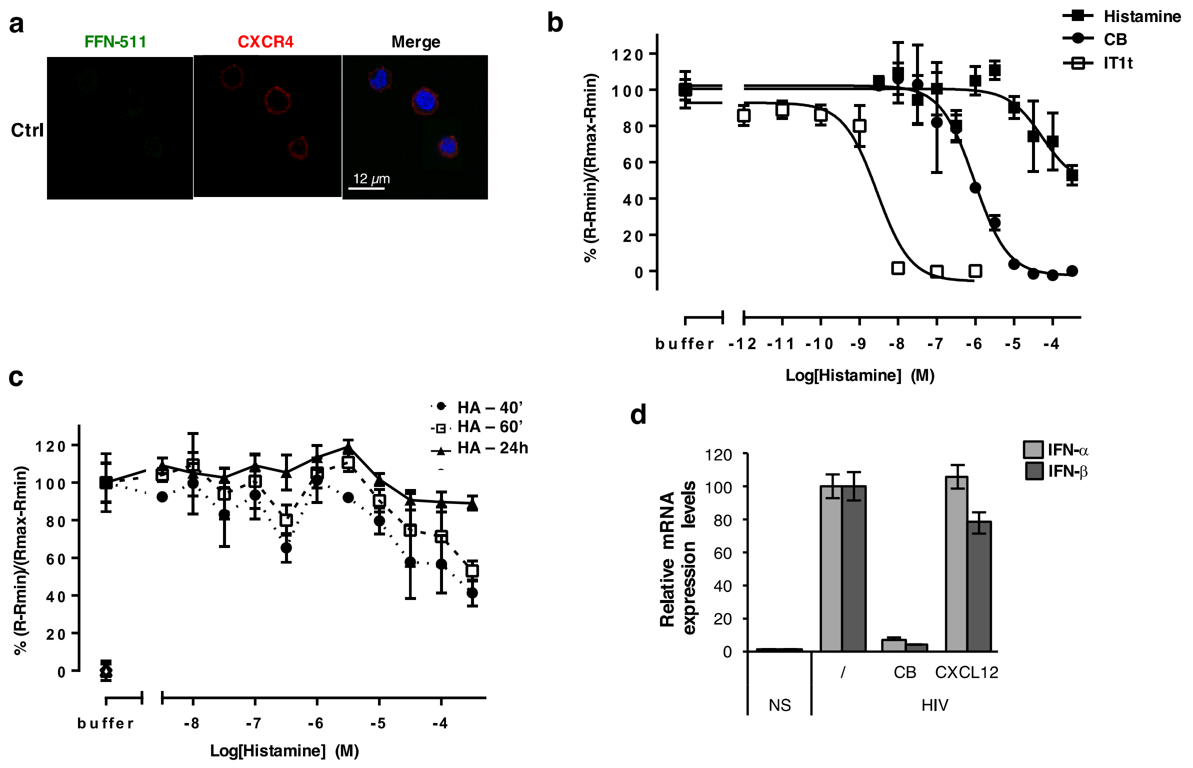

Supplementary Figure 3. The mechanism of amines inhibitory effect on HIV-stimulated pDC requires the binding to the chemokine receptor CXCR4. (a) Confocal microscopy of purified pDC. Cells were then fixed with formaldehyde, blocked with 0,5% BSA, stained with anti-CXCR4 (12G5) (red) and mounted with Fluoromount G with DAPI (blue). Scale bar, 12µm. **(b)** and **(c)** To evaluate the affinity of ligands for CXCR4 receptor, competition experiments were performed with the Tag-lite assay on HALOtag-CXCR4 receptors. Cells expressing HALO-tag CXCR4 were first incubated in the presence of HALOtag- Lumi4-Tb (100nM) for at least 1hour and incubated in the presence of CXCL12-red tracer (5nM) and increasing concentration of competitors. FRET signal were measured after 60 minutes **(b)** or 40 minutes, 60 minutes or overnight incubation **(c)** and fluorescence ratios (665 nm/620 nm) were plotted as a function of competitor concentrations. All binding data were analyzed using the one site-specific binding equation. Competition curves correspond to representative results of at least three independent experiments performed in triplicate. Ki values were calculated from IC50 values with the Cheng Prusoff equation 1. The non-specific binding was determined in the presence of an excess of IT1t (1µM). **(d)** mRNA levels of IFN-α/β from purified pDC pre incubated with CB (10µM) or CXCL12 (10µM) for 1h and stimulated overnight with HIV, were measured by RT qPCR and normalized to RPL13A. Error bars indicate the mean and SEM. Data shown are representative of three independent experiments.

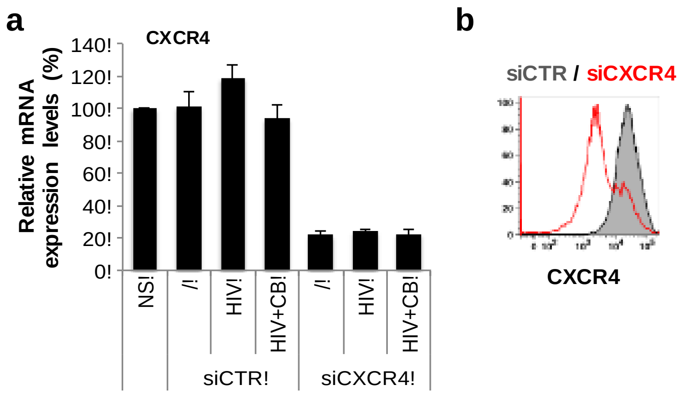

Supplementary Figure 4. The mechanism of amines inhibitory effect on viral-stimulated pDC requires CXCR4 expression. (a) mRNA levels of CXCR4 from purified pDC treated for 24h with a siRNA CXCR4 (siCXCR4) or a siRNA Control (siCTR) at 160nM and then pre-incubated with CB (10µM) and stimulated with HIV, were assessed by RT-qPCR and normalized to RPL13A. **(b)** Levels of membrane CXCR4 from purified pDC treated for 24hwith a siRNA CXCR4 (siCXCR4) or a siRNA Control (siCTR) at 160nM and then pre-incubated with CB (10µM) and stimulated with HIV were assessed by flow cytometry. Error bars indicate the mean and SEM. Data shown are representative of three independent experiments.

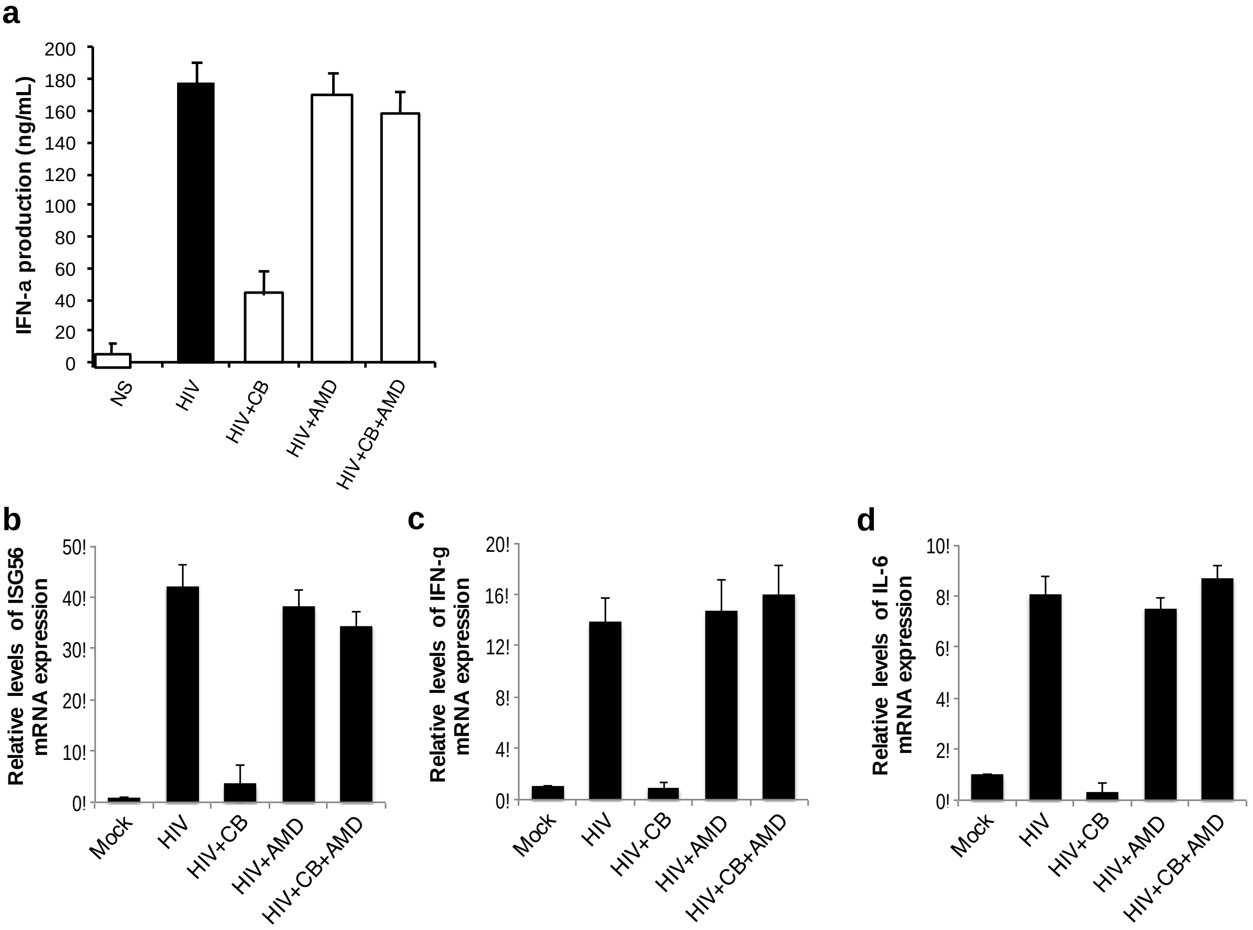

Supplementary Figure 5. The mechanism of amine inhibitory effect on viral-stimulated pDC requires the binding to the chemokine receptor CXCR4. (a) IFN-α was measured by ELISA in supernatants of isolated pDC pretreated with AMD3100 (20µM), then CB and stimulated with HIV overnight. **(b)**, **(c)** and **(d)** mRNA levels of ISG56 **(b)**, IFN-α/β **(c)** and IL-6 **(d)** from purified pDC pre-incubated with AMD-3100 (20µM) then CB (10µM) and stimulated overnight with HIV, were measured by RT-qPCR and normalized to RPL13A. Error bars indicate the mean and SEM. Data shown are representative of three independent experiments.

SUPPLEMENTARY METHODS

Chemical compound synthesis.

General methods

Tetrahydrofuran (THF) was distilled from sodium/benzophenone, immediately prior to use and stored under an argon atmosphere. 8-Hydroxyjulolidine, mono-*tert*-butyl succinate, potassium hydrogen methyl malonate, anhydrous dimethylformamide (DMF), ether and dioxane were purchased from Aldrich. *N*-*tert*-Butyloxycarbonyl-β-alanine was synthesized following literature procedure [1].

All reactions were carried out under argon atmosphere, and were monitored by thin layer chromatography with Merck 60F-254 pre-coated silica (0.2 mm) on glass. Flash chromatography was performed with Merck Kieselgel 60 (200–500 μm); the solvent systems were given v/v. ^{1}HNMR (500 MHz) and ^{13}C NMR (125 MHz) spectra were recorded on a Bruker AVANCEII-500 spectrometer. Chemical shifts (δ) are reported in ppm. The multiplicities are abbreviated as follows: s=singulet, d=doublet, t=triplet, m=multiplet, brs=broad singulet. ^{13}C chemical attributions were assigned using ^{1}H-decoupled spectra and 2D NMR experiments (HSQC, HMBC). Mass spectra were recorded on a Thermo Finnigan LCD Advantage spectrometer. Spectroscopic (^{1}H and ^{13}C NMR, MS) and/or analytical data were obtained using chromatographically homogeneous samples.

These studies were performed on a Nexera X2 UPLC instrument (Shimadzu, Marne La Vallée, France) coupled to a EXACTIVE Orbitrap mass spectrometer (Thermo, Les Ulis, France), using a ACE Excel 2C18-Amide column (150 × 2.1 mm, 2.3 μm), and a gradient A + B starting at 20% B for 5 min then increasing linearly to 100% B in 15 min (A = 10 mM ammonium acetate plus 0.1% HCOOH, pH 4.6, and B = CH3CN /HCOOH (999:1)) at 200 μL/min. Mass spectra were obtained by electrospray ionization (ESI) in positive ionization mode detection under the following conditions: source parameters, sheeth gas, 15; auxiliary gas, 5; spray voltage, 3.2 kV; capillary temperature, 275 °C; and m/z range for MS recorded generally between 150 and 900. For all products, the indicated parent ions corresponded to [M + H]+.

The synthesis of compounds **FFN-511**, **FC-CO2H** was achieved according to the following scheme:

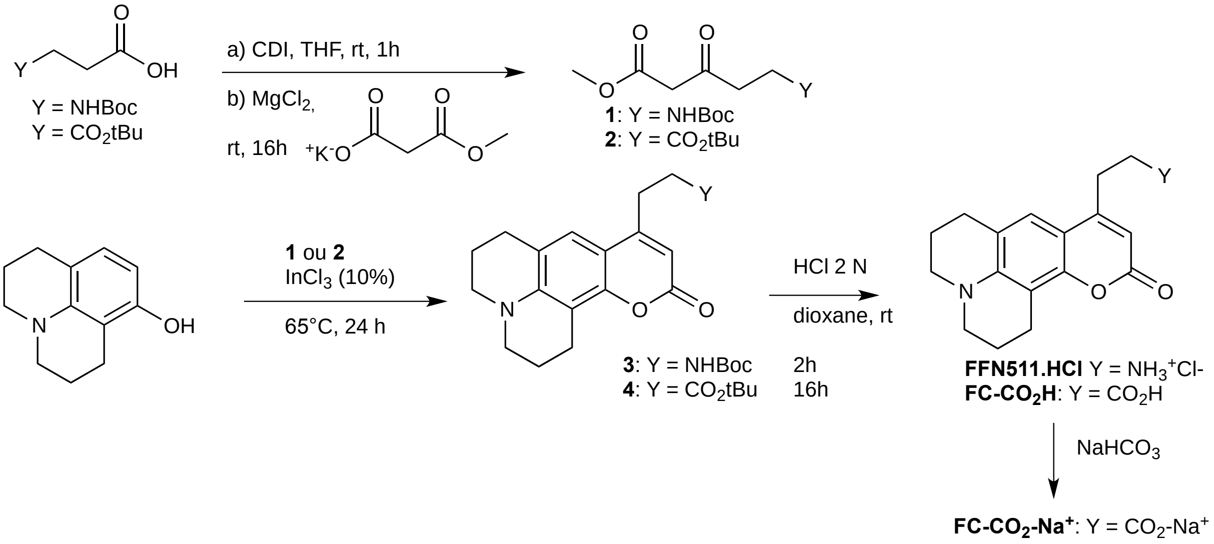

Procedures and Characterization of Products

General Procedure A: Masamune-Claisen type condensation [2]

1,1'-Carbonyldiimide (5.84 g, 36 mmol, 1.2 equiv) was added slowly to a solution of *N*-Boc-β-alanine (5.68 g, 30 mmol, 1 equiv) *or* mono-*tert*-butyl succinate (5.23 g, 30 mmol, 1 equiv) in anhydrous THF (100 mL) and the mixture was stirred at room temperature for 1 h. A well homogenized powdered solid mixture of $MgCl_2$ (2.74 g, 28.8 mmol, 0.96 equiv) and potassium hydrogen methyl malonate (7.03 g, 45 mmol, 1.5 equiv) was then added and the reaction mixture was stirred overnight. After evaporation of the solvent, EtOAc (100 mL) was added and the mixture was washed with 1 M aqueous $NaHSO_4$ (3 x 30 mL) and brine (30 mL). The organic phase was dried over $MgSO_4$, filtered and evaporated under reduced pressure. The residue was purified by flash chromatography on silica gel (cyclohexane/EtOAc) to give 6.55 g (89%) of **1** as a colorless oil [R_f= 0.3 (cyclohexane/EtOAc: 7/3)] *or* 5.87 g (85%) of **2** as a colorless oil [R_f= 0.3 (cyclohexane/EtOAc: 8/2)]. Spectral data of the β-keto esters **1** and **2** were in agreement with the literature data [3-5].

General Procedure B: Pechmann condensation [6]

A mixture of β-keto ester **1** (1 equiv) *or* **2** (1 equiv), 8-hydroxyjulolidine (1 equiv) and $InCl_3$ (0.1 equiv) was heated at 65 °C for 24 h. The cooled mixture was dissolved in $CHCl_3$, washed with water, dried over $MgSO_4$, filtered and evaporated under reduced pressure. The residue was purified by flash chromatography on silica gel (CH_2Cl_2/EtOAc).

General Procedure C: Removal of the *t*-butyl group

At 0 °C, to compound **3** *or* **4** was added 2 M HCl (20 equiv/*t*-Bu) in dioxane. The reaction mixture was then stirred at room temperature for 2–16 h and evaporated to dryness.

(2,3,5,6-Tetrahydro-1*H*,4*H*-10-oxo-11-oxa-3a-aza-benzo[*de*]anthracen-8-ylethylamino) methanoate *tert*-butyl ester (3). Following the *general procedure B*, β-keto ester **1** (981.1 mg, 4.0 mmol), 8-hydroxyjulolidine (757.0 mg, 4.0 mmol) and $InCl_3$ (85.6 mg, 0.4 mmol) yielded the title compound (800 mg, 52%) as a bright yellow solid; m.p. 78°C; [R_f= 0.32 (CH_2Cl_2/EtOAc: 9/1)].

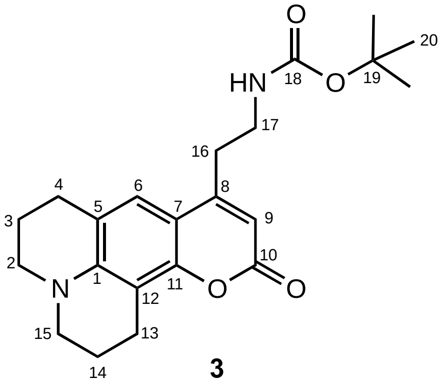

3

^{1}H NMR (500 MHz, $CDCl_3$): δ 7.0 (s, 1H, H-6), 5.84 (s, 1H, H-9), 4.72 (brs, 1H, NH), 3.38 (m, 2H, H-17), 3.21 (m, 4H, H-2, H-15), 2.83 (m, 4H, H-13, H-16), 2.73 (t, *J*= 6.5 Hz, 2H, H-4), 1.92 (t, *J*= 6.0 Hz, 4H, H-3, H-14), 1.40 (s, 9H, H-20); ^{13}C NMR (125 MHz, $CDCl_3$): δ 162.5 (C-10), 155.9 (C-18), 154.0 (C-8), 151.6 (C-11), 146.1 (C-1), 121.7 (C-6), 118.3 (C-5), 108.0 (C-7), 107.9 (C-9), 107.1 (C-12), 79.7 (C-19), 50.1 (C-2), 49.7 (C-15), 39.9 (C-17), 32.5 (C-16), 28.6 (C-20), 27.9 (C-4), 21.7, 20.8, 20.6 (C-3, C-13, C-14); MS, m/z (%): $[M+H]^+$= 385.5 (100%).

tert-Butyl (2,3,5,6-tetrahydro-1*H*,4*H*-10-oxo-11-oxa-3a-aza-benzo[*de*]anthracen-8-ylethyl) methanoate (4). Following the *general procedure B*, β-keto ester **2** (691.0 mg, 3.0 mmol),

8-hydroxyjulolidine (567.8 mg, 3.0 mmol) and $InCl_3$ (66.4 mg, 0.3 mmol) yielded the title compound (832.0 mg, 75%) as a yellow solid; m.p. 129 °C; [R_f= 0.34 (CH_2Cl_2/EtOAc: 95/5)].

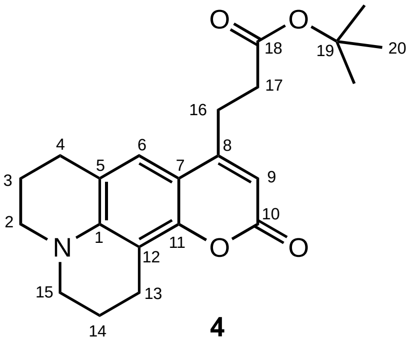

4

^{1}H NMR (500 MHz, $CDCl_3$): δ 6.90 (s, 1H, H-6), 5.76 (s, 1H, H-9), 3.15 (t, *J*= 5.5 Hz, 2H, H-2), 3.12 (t, *J*= 6.0 Hz, 2H, H-15), 2.83 (t, *J*= 7.5 Hz, 2H, H-16), 2.75 (t, *J*= 6.0 Hz, 2H, H-13), 2.67 (t, *J*= 5.5 Hz, 2H, H-4), 2.48 (t, *J*= 7.5 Hz, 2H, H-17), 1.85 (m, 4H, H-3, H-14), 1.36 (s, 9H, H-20); ^{13}C NMR (125 MHz, $CDCl_3$): δ 171.3 (C-18), 162.1 (C-10), 155.0 (C-8), 151.1 (C-11), 145.7 (C-1), 121.1 (C-6), 118.0 (C-5), 107.6 (C-7), 106.8 (C-12), 106.7 (C-9), 80.8 (C-19), 49.8 (C-2), 49.3 (C-15), 33.8 (C-17), 28.0 (C-20), 27.7 (C-4), 26.6 (C-16), 21.5 (C-3), 20.6, 20.4 (C-13, C-14); MS, m/z (%): $[M+H]^+$= 370.1 (100%).

8-(2-Aminoethyl)-2,3,4,5-tetrahydro-1*H*,4*H*-11-oxa-3a-aza-benzo[*de*]anthracen-10-one.HCl (FFN-511.HCl). The title compound was prepared following the *general procedure C* from **3** (192.2 mg, 0.5 mmol) after stirring for 2 h. Ether was then added to the residue and the precipitate was filtered. The precipitate was taken up in water and filtered then the filtrate was lyophilized to furnish **FFN-511.HCl** (154.0 mg, 96%) as a yellow solid.

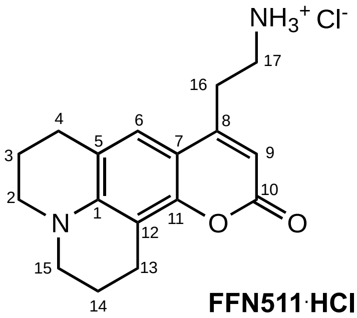

FFN511·HCl

^{1}H NMR (500 MHz, DMSO): δ 8.22 (brs, 3H, NH_3^+), 7.27 (s, 1H, H-6), 5.93 (s, 1H, H-9), 3.26 (t, *J*= 5.5 Hz, 2H, H-2), 3.24 (t, *J*= 5.5 Hz, 2H, H-15), 3.14-2.88 (m, 4H, H-16, H-17), 2.74 (t, *J*= 6.0 Hz, 2H, H-4), 2.72 (t, *J*= 6.5 Hz, 2H, H-13), 1.88 (m, 4H, H-3, H-14); ^{13}C NMR (125 MHz, DMSO): δ 160.6 (C-10), 152.1 (C-8), 150.8 (C-11), 145.6 (C-1), 121.6 (C-6), 117.9 (C-5), 107.5 (C-9), 106.7 (C-7), 105.7 (C-12), 49.2, 48.6 (C-2, C-15), 37.9 (C-17), 28.8 (C-16), 27.0 (C-4), 20.9, 20.1, 20.0 (C-3, C-13, C-14); MS, m/z (%): $[M–Cl^-]^+$= 285.1 (100%).

(2,3,5,6-Tetrahydro-1*H*,4*H*-10-oxo-11-oxa-3a-aza-benzo[*de*]anthracen-8-yl) propionic acid (FC-CO_2H). The title compound was prepared following the *general procedure C* from **4** (184.6 mg, 0.5 mmol) after stirring overnight. The residue was purified by flash chromatography on silica gel (CH_2Cl_2: 100 to CH_2Cl_2/MeOH: 9/1) to yield **FC-CO_2H** (139.5 mg**,** 89%) as a yellow solid; m.p. 144–146 °C; [R_f= 0.17 (CH_2Cl_2/ MeOH: 95/5)].

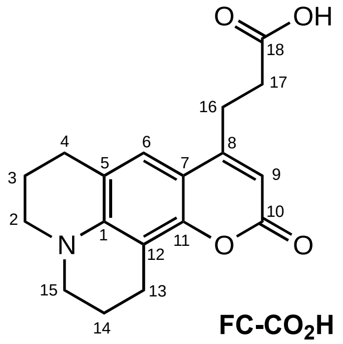

FC-CO_2H

^{1}H NMR (500 MHz, $CDCl_3$): δ 6.96 (s, 1H, H-6), 5.83 (s, 1H, H-9), 3.20 (m, 4H, H-2, H-15), 2.95 (t, *J*= 7.5 Hz, 2H, H-16), 2.83 (t, *J*= 6.5 Hz, 2H, H-13), 2.73 (t, *J*= 6.5 Hz, 2H, H-4), 2.63 (t, *J*= 7.5 Hz, 2H, H-17), 1.93 (m, 4H, H-3, H-14); ^{13}C NMR (125 MHz, $CDCl_3$): δ 172.7 (C-18), 162.5 (C-10), 154.8 (C-8), 151.4 (C-11), 146.0 (C-1), 121.2 (C-6), 118.3 (C-5), 107.8 (C-7), 107.1 (C-12), 107.0 (C-9), 50.1 (C-2), 49.6 (C-15), 32.7 (C-17), 27.9 (C-4), 26.7 (C-16), 21.7 (C-3), 20.8, 20.6 (C-13, C-14); MS, m/z (%): $[M+H]^+$= 314.1 (100%); $[M–H]^-$= 312.0 (100%), $[M–CO_2]^-$= 268.1 (50%).

FC-CO_2H was converted as its carboxylate sodium salt for water solubility: To **FC-CO_2H** in $CHCl_3$ under vigorous stirring was carefully added a 5% aqueous solution of $NaHCO_3$ (0.95 equiv).

After decantation the aqueous phase was lyophilized to yield **FC-$CO_2^-Na^+$**. MS, m/z (%): $[M+H]^+$= 314 with tr =15.51 min.

SUPPLEMENTARY REFERENCES

1 Salmon-Chemin, L. *et al.* 2-and 3-substituted 1,4-naphthoquinone derivatives as subversive substrates of trypanothione reductase and lipoamide dehydrogenase from Trypanosoma cruzi: Synthesis and correlation between redox cycling activities and in vitro cytotoxicity. *J Med Chem* **44**, 548-565, doi:Doi 10.1021/Jm001079l (2001).

2 Kralj, D. *et al.* A synthesis of 1-substituted 5-[2-(acylamino)ethyl]-1H-pyrazole-4-carboxamides. *Tetrahedron* **65**, 7151-7162, doi:Doi 10.1016/J.Tet.2009.06.021 (2009).

3 Moreau, R. J. & Sorensen, E. J. Classical carbonyl reactivity enables a short synthesis of the core structure of acutumine. *Tetrahedron* **63**, 6446-6453, doi:Doi 10.1016/J.Tet.2007.03.024 (2007).

4 Hashiguchi, S., Kawada, A. & Natsugari, H. Bakers-Yeast Reduction of N-Protected Methyl 4-Amino-3-Oxobutanoates and 4-Amino-3-Oxopentanoates. *Synthesis-Stuttgart*, 403-408 (1992).

5 Guzzo, P. R. & Miller, M. J. Catalytic, Asymmetric-Synthesis of the Carbacephem Framework. *J Org Chem* **59**, 4862-4867, doi:Doi 10.1021/Jo00096a031 (1994).

6 Gubernator, N. G., Sames, S., Sulzer, D. & Vadola, P. Fluorescent substrates for monoamine transporters as optical false neurotransmitters. Patent WO2008/013997 A2.
